# A novel strain-based bone-fracture healing algorithm is able to predict a range of healing outcomes

**DOI:** 10.3389/fbioe.2024.1477405

**Published:** 2024-10-18

**Authors:** George T. Morgan, Lucas Low, Arul Ramasamy, Spyros D. Masouros

**Affiliations:** ^1^ Department of Bioengineering, Imperial College London, London, United Kingdom; ^2^ Academic Department of Military Trauma and Orthopaedics, Royal Centre for Defence Medicine, ICT Centre, Birmingham, United Kingdom; ^3^ Trauma and Orthopaedics, Milton Keynes Hospital NHS Foundation Trust, Milton Keynes, United Kingdom

**Keywords:** fracture healing algorithm, bone, principal strains, fracture fixation, *in silico* trial, healing assessment, non-union

## Abstract

Fracture healing is a complex process which sometimes results in non-unions, leading to prolonged disability and high morbidity. Traditional methods of optimising fracture treatments, such as *in vitro* benchtop testing and *in vivo* randomised controlled trials, face limitations, particularly in evaluating the entire healing process. This study introduces a novel, strain-based fracture-healing algorithm designed to predict a wide range of healing outcomes, including both successful unions and non-unions. The algorithm uses principal strains as mechanical stimuli to simulate fracture healing in response to local mechanical environments within the callus region. The model demonstrates good agreement with experimental data from ovine metatarsal osteotomies across six fracture cases with varying gap widths and inter-fragmentary strains, replicates physiological bony growth patterns, and is independent of the initial callus geometry. This computational approach provides a framework for developing new fracture-fixation devices, aid in pre-surgical planning, and optimise rehabilitation strategies.

## 1 Introduction

Fracture healing is a complex process influenced by several mechanobiological parameters. In order to optimise fracture healing whilst taking into account these parameters, best treatment practices are determined typically by comparing treatments through *in vitro* benchtop testing and *in vivo* randomised controlled trials (RCTs). However, the debate over optimal surgical treatments for certain fractures, such as distal femoral fractures, persists due to limitations of *in vitro* testing and *in vivo* RCTs (Megafu et al., 2022; Wadhwa et al., 2022). *In vitro* testing of fracture fixation is constrained by the scope of bioreactor ossification, which is limited to early stages of callus formation (Hoffmann et al., 2015) or bony ingrowth and remodelling of *ex vivo* bone (Kohli et al., 2023). *In vitro* assessments are therefore limited to the initial fixation stiffness and preclude evaluation of the effects of loading on the healing process itself. *In vivo* testing through RCTs can be limited by challenges in patient and surgeon recruitment, especially in less common fractures, as demonstrated by a feasibility study which concluded that an adequately powered RCT for distal femoral fracture fixation would not be viable due to limited patient and surgeon recruitment (Griffin et al., 2019). Similar medical questions which have been left unanswered by traditional testing have recently been addressed effectively by *in silico* trials (Pappalardo et al., 2022; Pappalardo et al., 2019; Sarrami-Foroushani et al., 2021; Viceconti et al., 2021). Currently, there is no such *in silico* trial for fracture treatment as computational tools for fracture healing are under-developed.

Fracture-healing numerical algorithms have been developed that simulate fracture-healing progression in response to local mechanical environments in the callus region. These algorithms have previously been applied to the optimisation of fracture-fixator stiffness to improve healing outcomes (Nayak et al., 2024; Quinn et al., 2022; Steiner et al., 2014). Further potential applications of this technology include novel fracture-fixation device development, pre-surgical planning, and rehabilitation-regime optimisation. Previous fracture healing algorithms, however, have limited abilities to explore such applications adequately. While some algorithms have been compared against *in vivo* experimental data, they have generally only been shown to replicate successful healing cases; they have not been shown to predict cases resulting in non-union. This limits these algorithms from being used as predictive tools for non-union risk assessment and therefore pre-clinical decision making and improvement of fracture treatment.

The ability of existing algorithms to demonstrate a physiological healing sequence is dependent upon, and highly sensitive to, the diffusion-rate parameter, which represents either a process of cellular diffusion (Ghiasi et al., 2019; Isaksson et al., 2008; Lacroix and Prendergast, 2002; Quinn et al., 2022) or angiogenesis (Shefelbine et al., 2005; Simon et al., 2011). These processes require the presence of mesenchymal stem cells or vascularisation, respectively, in a specific region of the callus to allow ossification to occur. Diffusion is initialised from the external callus boundary, which prevents spurious bony union in the inter-cortical gap. This means that, in addition to these algorithms being highly sensitive to the diffusion rate parameter, they are also highly sensitive to the initial callus region geometry. As callus geometry is highly variable between fracture types and the callus shape is unknown at the point of pre-surgical planning, this limitation further precludes these algorithms from several possible applications. To address this limitation, a recent algorithm did not include a diffusion process and instead used a spatial proximity function which specifically prevented early ossification in the inter-cortical gap (Schwarzenberg et al., 2021b). This attempt, however, had the same effect in prescribing the callus ossification pathway as the algorithms which used a diffusion process.

**FIGURE 1:**
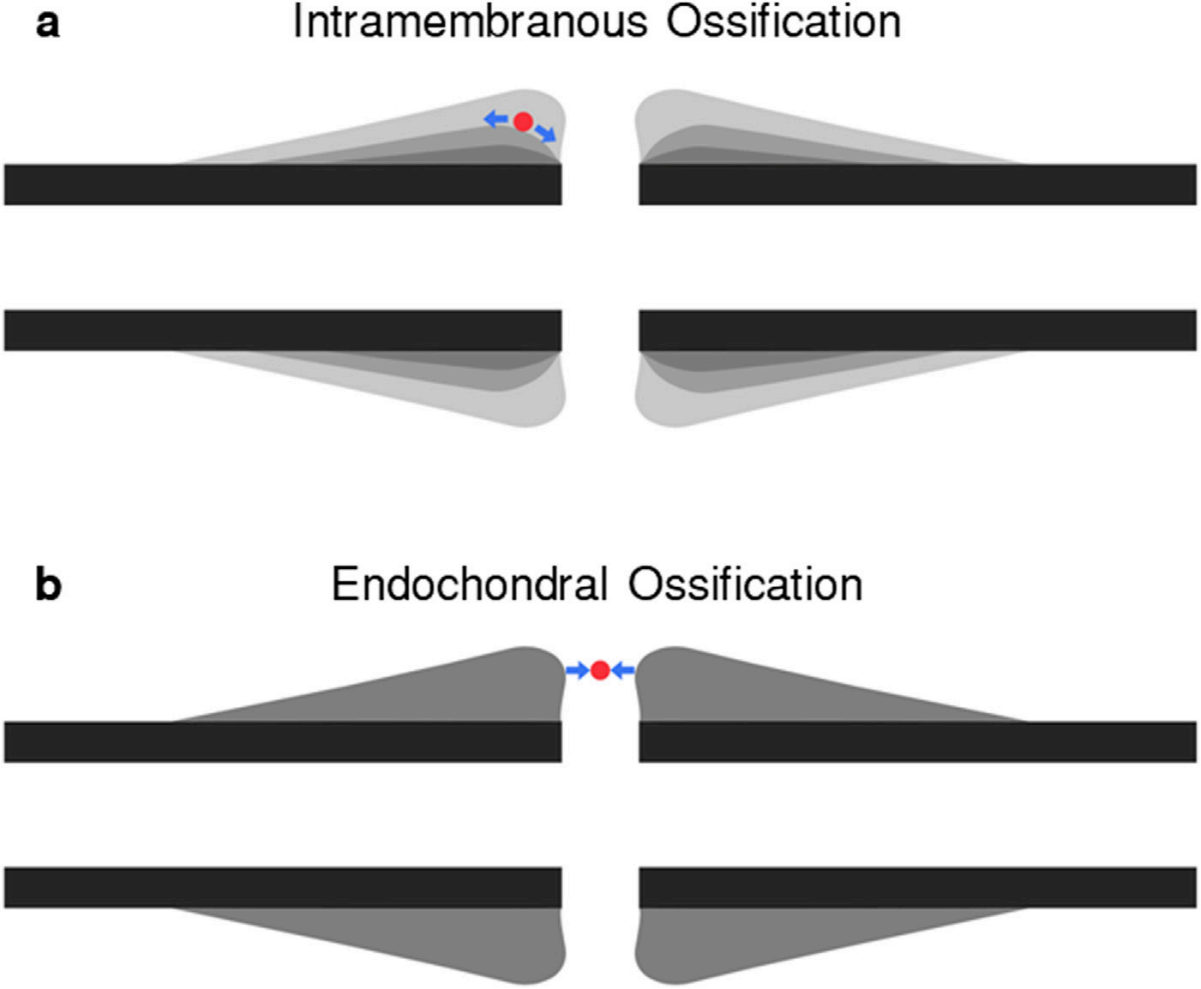
Diagram of **(A)** intramembranous ossification and **(B)** endochondral ossification pathways. In each panel, a representative “cell” laying on the ossification pathway is shown in red. During axial compression, the “cell” in panel **(A)** will experience shear, resulting in tensile strains, or high maximum principal strains, and in panel **(B)** will experience compressive strains, or high minimum principal strains.

All fracture healing algorithms which include strains as mechanical stimuli use the strain thresholds identified by Claes and Heigele (Claes and Heigele, 1999) for distortional and dilatational strain. Distortional strain has been identified as the more important strain input of the two in fracture-healing algorithms (Isaksson et al., 2006). While distortional strain is highly correlated to both minimum and maximum principal strains based on its mathematical formulation, it is not possible to determine the greater of the magnitudes of minimum and maximum principal strain based on the value of distortional strain. Therefore, distortional strain is an insufficient description of the strain state of the callus in fracture-healing modelling. During secondary fracture healing, intramembranous ossification occurs on the growing bony bulges on the periosteum where shear dominates, however, the material directly external to the bulges experiences tension, which can be represented with maximum principal strain (Figure 1). Chondrogenesis and endochondral ossification occur in the region between the bulges where compression dominates, and this can be represented by the magnitude of minimum principal strain. Therefore, the main processes of secondary fracture-healing can be predicted by the local minimum and maximum principal strains in the callus region. In this study, a novel fracture-healing algorithm, which is the first to use minimum and maximum principal strains as the mechanical stimuli, is presented and tested for validation against *in vivo* experimental data of cases which resulted in both successful unions and non-union.

## 2 Materials and methods

### 2.1 Fracture healing algorithm

**FIGURE 2:**
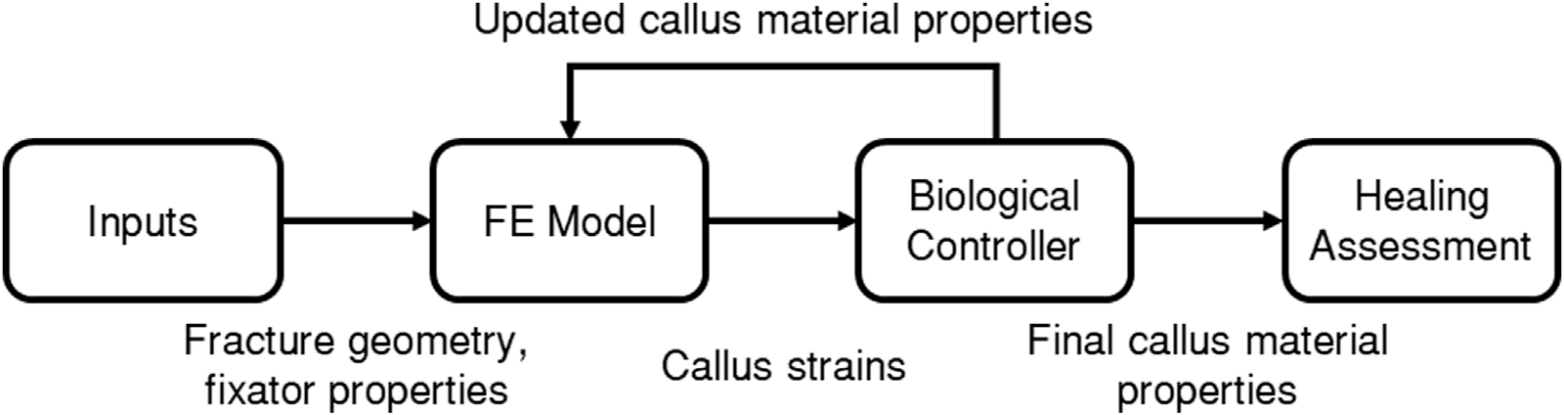
Flowchart of the fracture healing algorithm.

The fracture-healing algorithm developed and used in the present study is described by the flowchart in Figure 2, where each of the boxes “FE Model”, “Biological Controller”, and “Healing Assessment” are described in more detail below. A finite-element (FE) model was developed of the fracture site, the initial callus domain, and the fixator. Each callus finite-element was represented as a mixture of 3 tissue types: woven bone, cartilage, and soft tissue. Axial loading was applied and the resultant callus element strains were used as inputs to the biological controller, which determined updated tissue-type proportions, and in turn updated the callus element material properties in the FE model. This process was performed iteratively 150 times. The final FE model was then adapted to simulate a bending test to assess the bending stiffness of the final callus.

Six different fracture cases were modelled with initial gap widths of 1 mm, 2 mm, and 6 mm, and initial allowed inter-fragmentary strains (IFS) of 7% and 31%. Gap width is the distance between the two bone segments in the compressed state (Claes et al., 1997). IFS is the amount of axial displacement of the bone segments relative to each other divided by the initial gap width. The combination of gap widths and initial IFS for each case is given in Table 1. These 6 models correspond to the groups used in an *in vivo* experimental study on ovine metatarsal osteotomies stabilised with a custom external fixator (Claes et al., 1997), therefore allowing comparison between simulation results and experimental observations. The experimental groups included groups which resulted in successful unions and groups which resulted in non-unions.

**TABLE 1:** Fracture gap width and initial inter-fragmentary strain (IFS) in each of the simulated groups, corresponding to the groups used in an experimental study by Claes et al.

Group	A	B	C	D	E	F
Gap Width (mm)	1	1	2	2	6	6
IFS (%)	7	31	7	31	7	31

### 2.2 FE model

**FIGURE 3:**
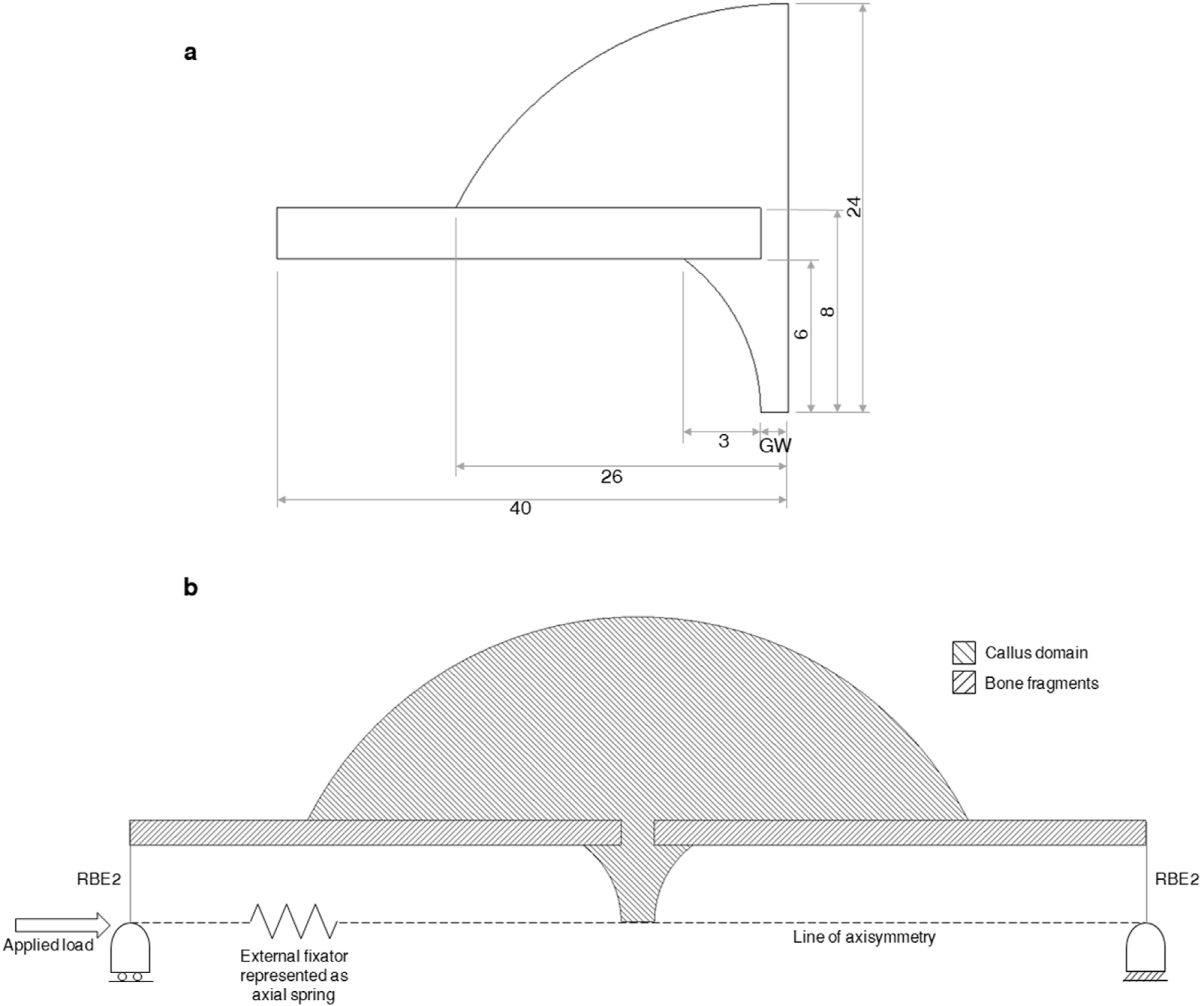
**(A)** Dimensions (in mm) of the bone fragments and callus region. GW denotes “gap width” in the uncompressed state and equals 1.07, 1.31, 2.14, 2.62, 6.42, and 7.86 mm for groups A-F, respectively. The gap widths of 1, 2, and 6 mm reported by Claes et al. were measured in the compressed state and must be scaled by the allowed initial interfragmentary strain of 7% or 31% to determine the gap width in the uncompressed state. **(B)** Diagram of the axisymmetric FE model of a fracture, callus domain, and fixator. The external fixator is represented as a non-linear axial spring running from the far ends of both bone fragments. The securement of the fixator to each bone section is represented with rigid-body-elements (RBE2’s) connecting the far end of each bone fragment to a node located on the line of axisymmetry. Boundary and loading conditions are shown. The callus domain is initialised with soft tissue material properties and the bone fragments are modelled as lamellar bone.

An axisymmetric non-linear FE model was developed in MSC.Marc (v2021, MSC Software) of a simple transverse mid-diaphyseal metatarsal ovine osteotomy secured with an external fixator. The callus was modelled explicitly (Figure 3A). An 80 mm long section of the fracture region was modelled with the metatarsus represented as a hollow cylinder, with outer diameter and thickness of 16 mm and 2 mm, respectively. The callus domain was initialised with a diameter of 48 mm and a length of 52 mm, and shaped according to a standardised callus domain geometry in fracture healing algorithms (Claes and Heigele, 1999; Shefelbine et al., 2005; Simon et al., 2011). The gap width of 1 mm, 2 mm, or 6 mm, references the gap width in the loaded state, as described in the experimental study by Claes et al. (1997). The geometry was meshed using linear triangular elements with an average element edge-length of 0.35 mm.

The tissue types modelled were soft tissue, cartilage, woven bone, and lamellar bone. These were assigned Young’s moduli of 3, 200, 4,000, and 10,000 MPa, respectively, and Poisson’s ratios of 0.30, 0.45, 0.36, and 0.36, respectively (Ren and Dailey, 2020; Simon et al., 2011). All materials were modelled as linearly elastic and isotropic (Ren and Dailey, 2020; Simon et al., 2011). All callus elements were assigned soft tissue material properties at the first iteration. After each iteration, the Young’s modulus and Poisson’s ratio of each callus element were calculated from the updated proportion of each tissue type within the element according to a cubic, and linear rule of mixture, respectively (Simon et al., 2011), as shown in Equations 1, 2, where $E_{element}$ and $\nu_{element}$ are the Young’s modulus and Poisson’s ratio of the element, $E_{tissue}$ and $\nu_{tissue}$ are the Young’s modulus and Poisson’s ratio of a tissue type, and $c_{element,tissue}$ is the concentration of the tissue in the element, where the expressions $E_{tissue}c_{element,tissue}^{3}$ and $\nu_{tissue}c_{element,tissue}$ are summed over all 3 tissue types allowed in the callus region: soft tissue, cartilage, and woven bone.

$$E_{element}=\sum_{tissue}E_{tissue}c_{element,tissue}^{3}$$ (1)

$$\nu_{element}=\sum_{tissue}\nu_{tissue}c_{element,tissue}$$ (2)

The external fixator used in Claes et al. was designed to allow for a pre-determined amount of axial motion (Claes et al., 1995). The amount of free axial movement allowed was set to the desired distance while the fixated limb was in the loaded state (Claes et al., 1997). In the present study, the fixator was modelled as a non-linear axial spring, with an initial stiffness of 4600 N mm^−1^ up to a force of 100 N, then a stiffness of 10 N mm^−1^ up to the desired free movement distance, and a stiffness of 4600 N mm^−1^ beyond (Claes et al., 1997; Simon et al., 2011).

Boundary conditions were applied at both ends of the bone construct (Figure 3). Nodes on the distal cut-face were held in three translational directions and nodes on the proximal cut-face were held in two translational directions, allowing for relative axial movement. These conditions were applied in FE by using rigid-body-elements (RBE2s) with a control node for each face placed on the line of axisymmetry. Ovine metatarsals experience predominantly axial loading (Duda et al., 1997) and the shear and torsional rigidities of the fixator are reported to be high, allowing for only axial movement (Claes et al., 1995; Claes et al., 1997; Claes and Heigele, 1999), therefore only axial loading was modelled, allowing the use of an axisymmetric modelling approach. The spring representing the external fixator connected the two retained nodes of the RBE2s at the bone ends. An axial load of 500 N was applied to the proximal retained node (Claes and Heigele, 1999; Simon et al., 2011).

### 2.3 Biological controller

A fuzzy logics approach was used to simulate some of the biological processes involved in secondary fracture healing based on previous fracture-healing algorithms (Ament and Hofer, 2000; Schwarzenberg et al., 2021b; Shefelbine et al., 2005; Simon et al., 2011), but using minimum and maximum principal strains as mechanical inputs for the first time. The biological fuzzy logic controller was developed using the Scikit-Fuzzy (v0.4.2) fuzzy logic toolbox (Warner et al., 2019) in Python (v3.6, Python Software Foundation). The fuzzy logic controller determines the proportion of soft tissue, cartilage, and woven bone in each finite-element at the next iteration. The inputs to the fuzzy controller are the minimum principal, maximum principal, and distortional strains in each element in the current iteration, as well as the proportion of cartilage and woven bone in each element $\left(c_{\text{cart}}\text{ and }c_{\text{bone}}\right)$ and the maximum proportion of cartilage and bone of all neighbouring elements $\left(c_{\text{nCart}}\text{ and }c_{\text{nBone}}\right)$ . Neighbouring elements are defined as elements which share at least 1 node. The proportion of soft tissue in each element $\left(c_{\text{soft}}\right)$ is determined from the relation given in Equation 3.

$$c_{soft}=1-c_{cart}-c_{bone}$$ (3)

**FIGURE 4:**
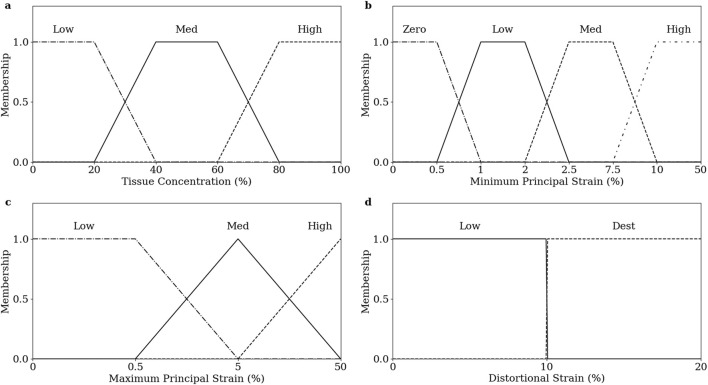
Fuzzy membership functions of inputs, **(A)** tissue prop, **(B)** minimum principal strain, **(C)** maximum principal strain, and **(D)** distortional strain.

Inputs are “fuzzified” by determining their membership of linguistic categorisations in their corresponding membership functions. The degree of membership is calculated on a 0 to 1 scale. Fuzzy membership functions were defined for tissue proportions (Figure 4A), minimum principal strain (Figure 4B), maximum principal strain (Figure 4C), and distortional strain (Figure 4D).

Four of the biological processes involved in the fracture healing process were implemented: chondrogenesis, cartilage calcification, intramembranous ossification, and endochondral ossification. Chondrogenesis was disabled for the first 7 iterations to prevent non-physiological periosteal cartilage formation. Each biological process was modelled as a fuzzy logic rule; the set of rules is given in Table 2.

**TABLE 2:** The four fuzzy logics rules implemented in the fracture healing algorithm.

Rule	Bone	Cart	nBone	nCart	Minimum principal strain	Maximum principal strain	Distortional strain	ΔBone	ΔCart
Intramembranous Ossification		Low	Med or High			Med	Low	1	0
Chondrogenesis	Low		Med or High		Med	Low	Low	0	1
Chondrogenesis	Low			Med or High	Med	Low	Low	0	1
Cartilage Calcification	High	Low	High					1	−1
Endochondral Ossification		Med or High	Med or High		Low			1	−1

The degree of activation of each rule was determined by interpreting the linguistic rule numerically. The membership of each individual statement was a value between 0 and 1, as determined by their input value and corresponding membership function. Each rule yields a single value between 0 and 1 which represents the degree of activation of that rule for the element. The effects on bone and cartilage concentration of all rules were summed for each element to determine the overall change in composition for the element. A temporal smoothing function was applied in which the bone and cartilage concentrations in the current iteration were calculated as the mean un-smoothed concentrations calculated by the algorithm on the previous N iterations (Lacroix and Prendergast, 2002). A parameter coefficient was used to scale the changes in bone and cartilage concentrations according to the element size. This parameter and the temporal smoothing parameter N were used to achieve mesh convergence. The results of a mesh convergence study are given in Supplementary Material.

### 2.4 Healing assessment

Bending stiffness of the fracture site was used as the healing assessment as IFM is a poor indicator of union (Ren and Dailey, 2020) and the corresponding experimental data includes a final bending stiffness assessment (Claes et al., 1997). Each axisymmetric FE model was converted to its corresponding 3D FE model to assess the bending stiffness of the final construct and resultant callus. The bone fragments were extended axially to create a 150 mm long fracture region, as was used in the experimental bending stiffness assessment by Claes et al. (1997). The bone fragments and callus were meshed with linear tetrahedral elements with element sizes of 1 mm and 0.5 mm, respectively. Material properties for the callus elements were mapped from the 2D callus by linearly interpolating values of Young’s modulus and Poisson’s ratio from the centroids of the 2D callus elements, or by using a “nearest” extrapolation for 3D callus elements whose centroids fell outside of the interpolation window created by the 2D callus element centroids. The cylindrical coordinates of the 3D callus-element centroids were mapped to the 2D coordinate system for this interpolation. This process was automated. The spring representing the external fixator was removed, as the fixator was explanted prior to experimental bending stiffness assessment (Claes et al., 1997). A bending moment of 1500 N mm was applied through the retained node of the RBE2s at each bone fragment end. The distal retained node was fixed in all 5 other degrees of freedom, and the proximal retained node was fixed in 4 degrees of freedom, allowing for axial translation. Deflection was measured at the central-most node of the callus. Bending stiffness was calculated as the applied moment divided by the mid-span deflection.

### 2.5 Code availability

The code used to implement the fracture healing algorithm in this study is available at https://github.com/GeorgeTMorgan/fracture-healing-algorithm.

## 3 Results

### 3.1 Effect of initial callus domain size

**FIGURE 5:**
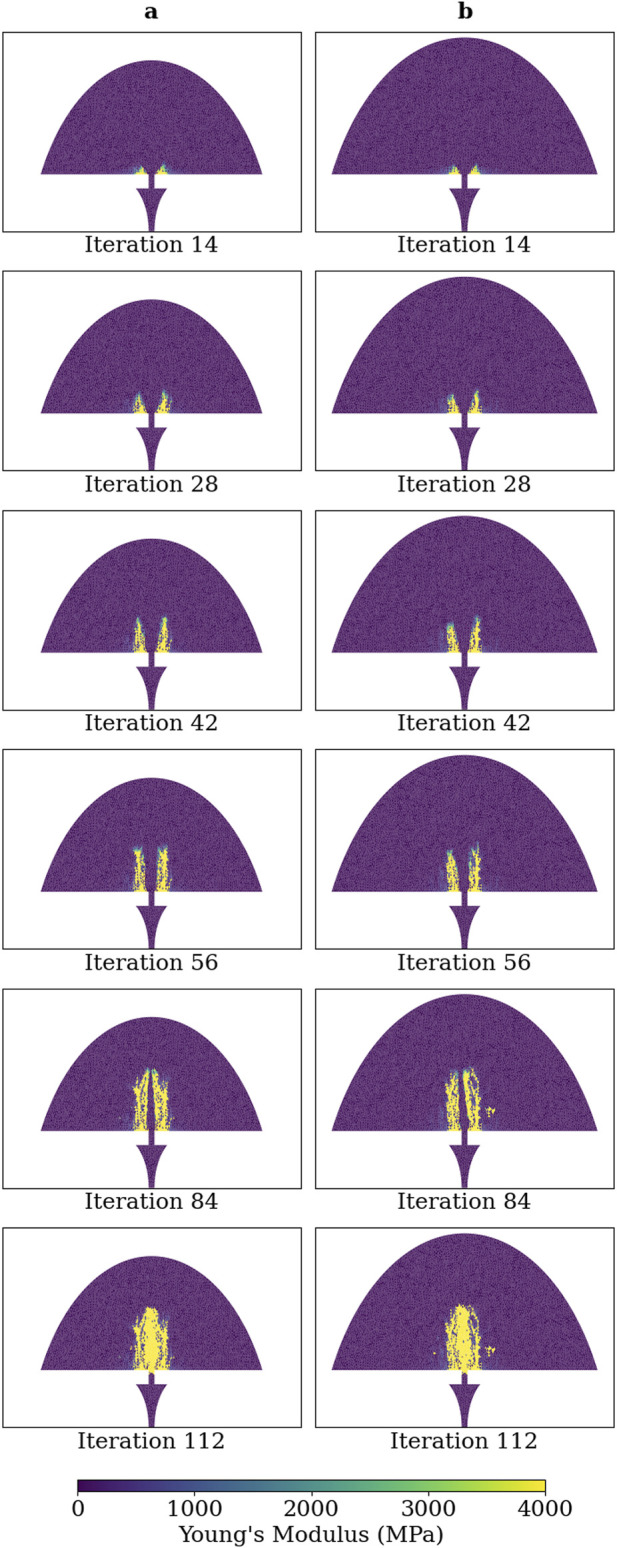
Young’s modulus of each callus finite-element in simulations of group B at iterations: 14, 28, 42, 56, 84, and 112. **(A)** Callus domain geometry initialised as described in Figure 3A and **(B)** callus domain initialised with radius and length increased by 13% and 20%, respectively.

No difference was observed on the qualitative progression on the simulated healing for Group B when increasing the initial region of the callus by 13% in radius and 20% in length, thereby demonstrating callus domain independence (Figure 5). The convergence of the healing simulation, defined by a change in IFM of less than 1% from the previous iteration, was delayed by 4 iterations in the simulation with a larger callus domain size.

### 3.2 Algorithm-predicted sequence of healing

**FIGURE 6:**
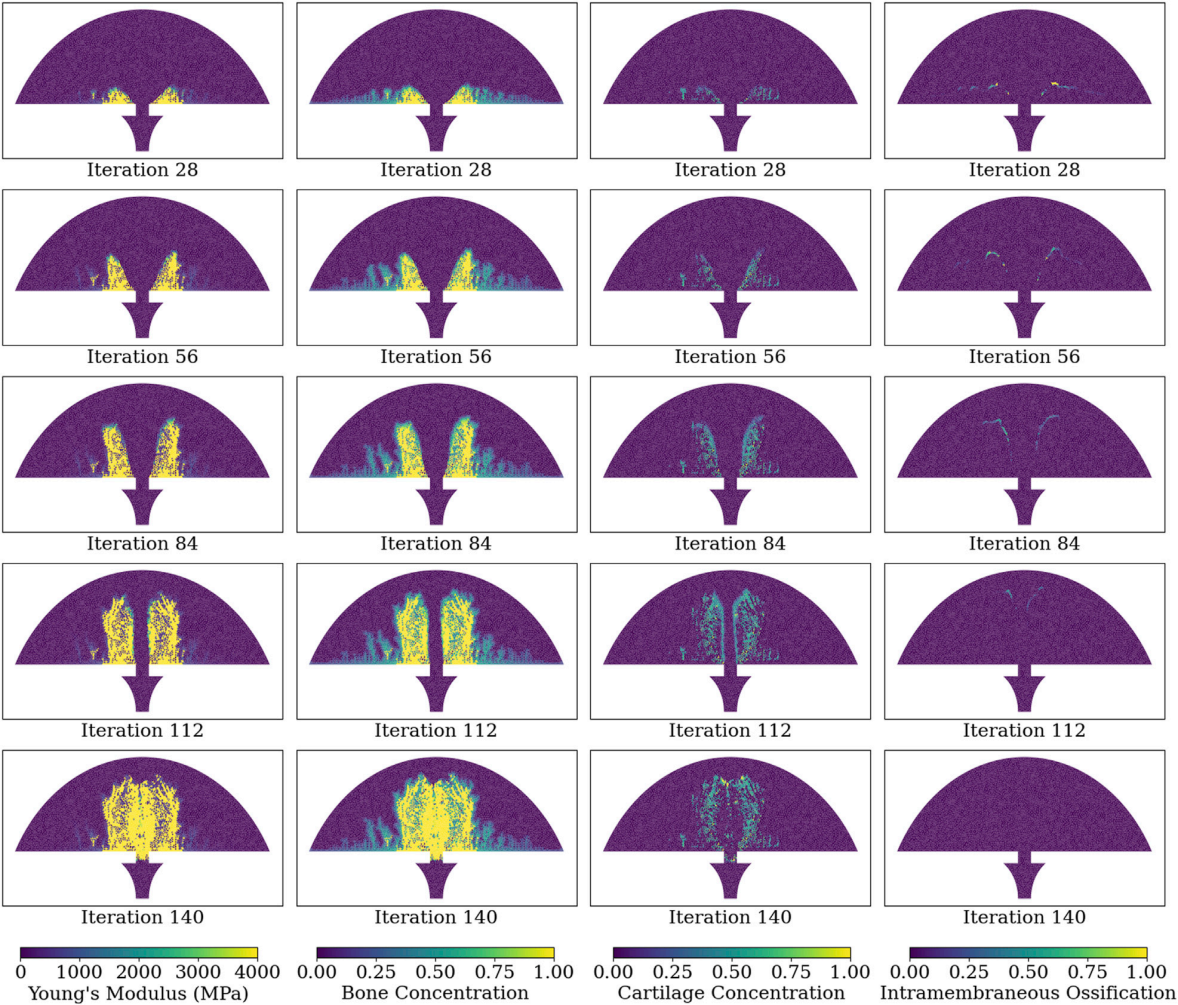
Simulated healing dynamics of Group D at iterations: 28, 56, 84, 112, and 140. The plotted variables for each callus finite-element span from the leftmost to the rightmost column: Young’s modulus, bone concentration, cartilage concentration, and intramembranous ossification activation. Bone and cartilage concentrations are depicted as tissue proportions within the finite-element, with a bone concentration of 1.0 indicating full ossification. Intramembranous ossification is quantified as an activation proportion, with a value of 1.0 denoting complete biological rule activation.

Figure 6 illustrates the Young’s modulus, bone concentration, cartilage concentration, and intramembranous ossification activation level at each callus finite-element during iterations 28, 56, 84, 112, and 140 of the simulation of group D. Notably, the healing algorithm demonstrates physiological bony growth on the periosteum via intramembranous ossification, with bridging occurring at the outer callus, rather than in the direct inter-cortical gap. The bridging process involves cartilage formation via chondrogenesis, and subsequent ossification through endochondral ossification, rather than intramembranous ossification.

### 3.3 Algorithm convergence

**FIGURE 7:**
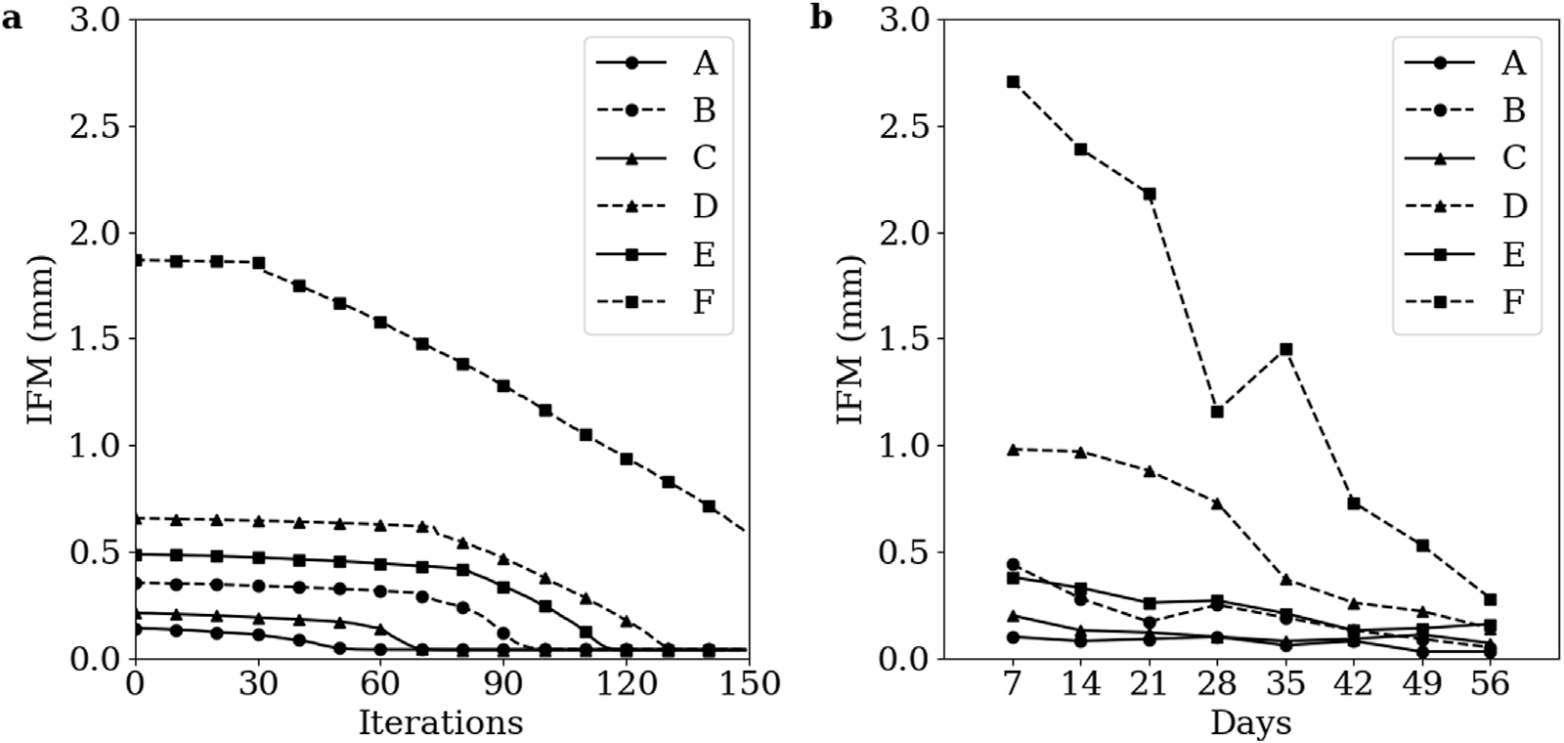
Temporal evolution of IFM across the healing process for groups A-F **(A)** Simulation data. **(B)** Corresponding experimental data. Groups A-F are as defined by Claes et al. The low strain groups A, C, and E are denoted with solid lines and the high strain groups B, D, and F are denoted with dashed lines. The 1 mm, 2 mm, and 6 mm initial gap size groups are denoted with circle, triangle, and square markers, respectively. Markers in the simulation data are denoted every 10 iterations.

Simulations of groups A-E converged at iterations 55, 102, 73, 136, and 120, respectively. The simulation of group F failed to converge within 150 iterations. The IFM progressions of each group are visually represented in Figure 7A, while the mean IFM progressions for each corresponding experimental group are displayed in Figure 7B (Claes et al., 1997). Notably, the IFM progressions in the simulated groups closely mirror those observed in the experimental groups, and indicate that one healing day in the experimental data corresponds approximately to three simulation iterations.

### 3.4 Virtual mechanical testing

Virtual mechanical testing, conducted at the conclusion of the 150 iterations on the corresponding 3D FE model, measured bending stiffnesses of 6.9, 7.3, 7.2, 8.4, 7.8, and 1.8 N m mm^−1^ for groups A through F, respectively. The corresponding experimental bending stiffnesses were 24.3 ± 7.6, 35.6 ± 23.1, 26.6 ± 18.6, 15.8 ± 14.6, 8.9 ± 7.0, and 1.6 ± 0.9 N m mm^−1^, respectively (Claes et al., 1997).

### 3.5 Healing sequence across groups

**FIGURE 8:**
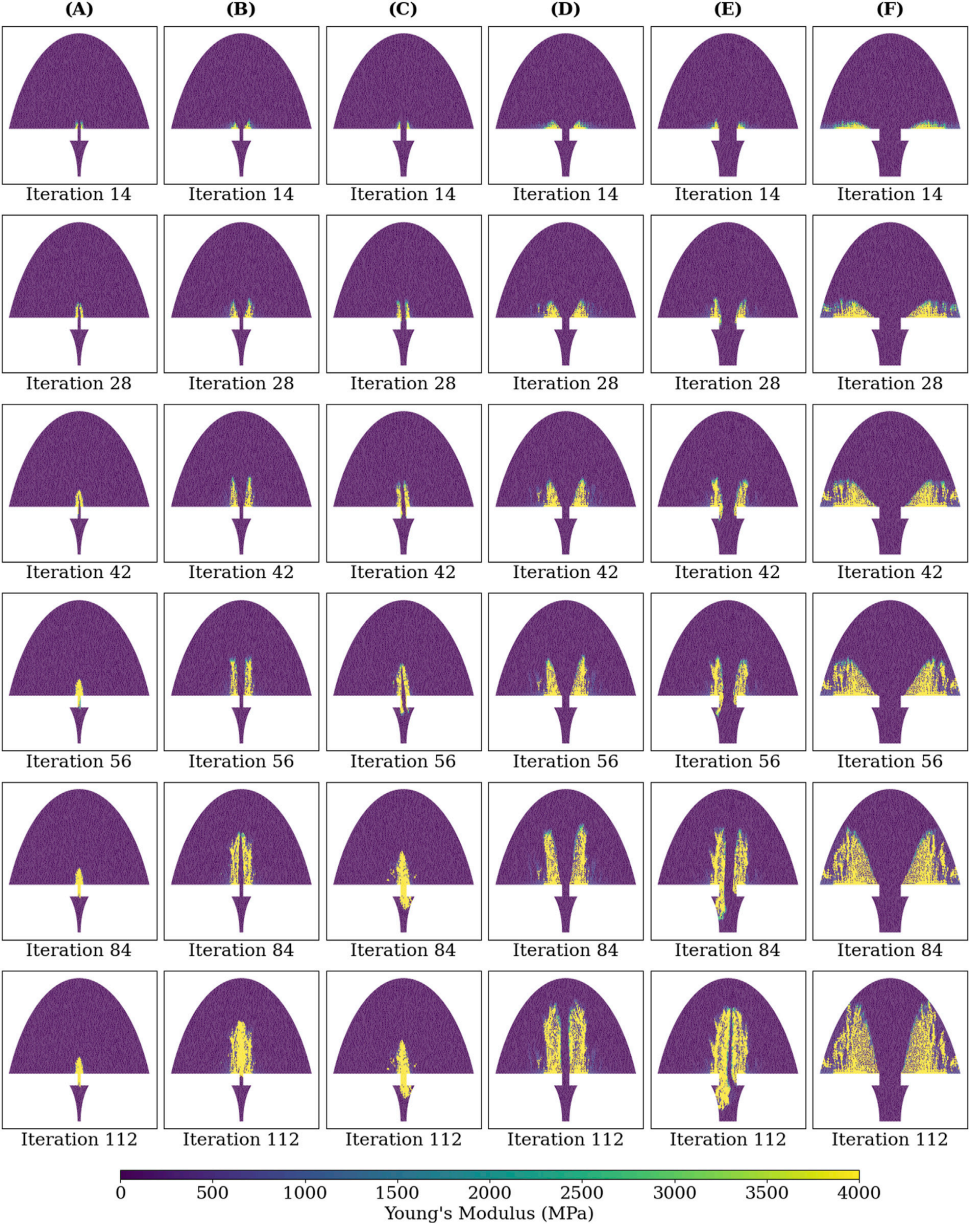
Young’s modulus of each callus finite-element at iterations: 14, 28, 42, 56, 84, and 112 of the simulated healing process for groups **(A-F)**.

Figure 8 shows the Young’s modulus at each callus finite-element during iterations 14, 28, 42, 56, 84, and 112 for groups A-F. The higher strain groups B, D, and F exhibited more extensive bony callus development compared to the lower strain groups A, C, and E. However, bony bridging in the higher strain groups was delayed compared to their lower strain counterparts.

## 4 Discussion

This study introduced a novel fracture-healing algorithm based on principal strains as the primary mechanical stimuli. The algorithm was able to match closely experimental observations across different fixation stabilities and initial fracture-gap widths. Notably, this algorithm is the first of its kind capable of predicting cases not only of fracture union, but also non-union. There is no clear definition of non-union in the literature; non-union is characterised by a failure to restore the physiological function of the bone, such as weight-bearing in lower-limb long bones. Non-unions are diagnosed radiographically by observing a lack of bony bridging (Litrenta et al., 2015; Whelan et al., 2010); they are quantified *in vitro* using mechanical testing (Claes et al., 1997), and *in silico* using virtual mechanical testing (Ren et al., 2024; Schwarzenberg et al., 2021a). This study used virtual mechanical testing of bending stiffness to quantify bony union and non-union. The ability to distinguish between scenarios which are likely to result in union *versus* non-union is a primary clinical application of fracture-healing algorithms as early predictors of non-union.

Intramembranous ossification is stimulated by shearing between the periosteum of the bone fragments and the soft callus region, with areas closer to the fracture experiencing higher shear forces and greater bony outgrowth. These characteristics of intramembranous ossification are effectively captured by maximum principal strain, as used in this fracture-healing algorithm. Chondrogenesis and endochondral ossification occur between the bony protrusions where compression, represented by minimum principal strain, dominates. Previous fracture-healing algorithms utilised porous flow (Ghiasi et al., 2019; Isaksson et al., 2008), dilatational strain, and distortional strain (Isaksson et al., 2006; Shefelbine et al., 2005; Simon et al., 2011), with distortional strain identified as the dominant mechanical stimulus (Isaksson et al., 2006). Although distortional strain is closely correlated with both minimum and maximum principal strains, it cannot distinguish between the two. The use of principal strains as mechanical stimuli in this healing algorithm enabled a physiological fracture-healing process.

The patterns of IFM progression across the six groups align well with the corresponding experimental data (Figure 7). The healing algorithm was not temporally calibrated prior to the analyses conducted in this study, but the IFM progression patterns in Figure 7 suggest that three simulated healing iterations correspond approximately to 1 day of healing. While the ability of the healing algorithm to predict similar IFM progression patterns as experimental data is encouraging, the IFM itself is not an accurate measure of fracture healing. This inaccuracy is exemplified by Group F in both the simulated and experimental results of Claes et al. (1997), where IFM decreases significantly over the course of healing, yet union is not achieved. Instead, more informative measures of successful fracture union should be used, such as bending stiffness or torsional rigidity (Ren and Dailey, 2020).

The bending stiffness assessments of simulated healing cases using this healing algorithm revealed that the healing algorithm could distinguish between groups that clearly healed (groups A-D) and those that did not (group F), mirroring the trends in bending stiffness among the corresponding groups in experimental data (Claes et al., 1997). Although the trend in bending stiffness between the different groups was similar in the healing algorithm and the experimental data, a limitation of the current study is the discrepancy in the magnitudes of bending stiffness. For instance, the bending stiffness of group A was greater than that of group F in both the healing algorithm and experimental results, but the magnitudes of bending stiffness for group A were 6.9 and 24.3 N m mm^−1^ for simulation and experiment, respectively. This discrepancy is likely due to the absence of bone remodelling in the healing algorithm; woven bone does not get replaced with stiffer lamellar bone, in a process that physiologically occurs from early-on in the secondary fracture-healing process (Ren and Dailey, 2020). Incorporating a remodelling process into the fracture-healing algorithm would likely further ossify the periosteally formed bone, which is further from the fracture, and in the current model does not achieve a bone concentration greater than approximately 30% (Figure 6). Another limitation of the current healing algorithm is its deterministic nature in simulating of “borderline” healing cases; the algorithm predicted successful fracture union in group E, whereas experimental data showed group E to have a mean bending stiffness between those of successful healing groups A-D and the unsuccessful healing group F. The large variance in the experimental bending stiffnesses, with a standard deviation of 7.0 N m mm^−1^ and a mean of 8.9 N m mm^−1^ in group E, suggests that rather than all of the sheep healing partially, likely some of the sheep in the group healed successfully while others experienced non-unions. This group represents an ambiguous healing case in which the specifics of loading, fracture geometry, and fixator stiffness are likely required to predict accurately the healing outcome, rather than the idealised parameters which were used in this study.

A significant advantage of the fracture-healing algorithm presented in this study is its independence from the initial callus domain and the absence of a pre-defined hard callus geometry, while exhibiting physiologically accurate stages of secondary fracture healing. The simulations for all successful healing groups began with periosteal bony outgrowth followed by cartilaginous, and later bony, initial bridging of the fracture gap at the external callus, and importantly, not in the intercortical gap. These qualitative stages of secondary fracture healing are in agreement with established literature (Claes et al., 2012; Iwaki et al., 1997; Vetter et al., 2010). Previous fracture-healing algorithms have either shown inter-cortical bridging (Ament and Hofer, 2000; Ren and Dailey, 2020) or enforced external callus bridging using a pre-defined spatial proximity function which inhibits bone formation in the intercortical gap (Schwarzenberg et al., 2021b). Other algorithms prevent inter-cortical bridging through a “cellular diffusion” (Isaksson et al., 2006; Lacroix and Prendergast, 2002) or “vascular perfusion” (Shefelbine et al., 2005; Simon et al., 2011) parameter, which begins at the outer surface of the callus and diffuses inwards, only allowing bone formation where this parameter is active. Algorithms which use this diffusion parameter are therefore highly sensitive to the initial callus domain and diffusion rate (Isaksson et al., 2009). Given that callus geometry is highly variable between fracture types, and that the callus does not form until after surgical intervention, a fracture-healing algorithm that relies on accurate prescribed callus geometry may be useful for post-surgical rehabilitation management and early diagnosis of non-union, but is less suited for pre-operative planning, novel device development, or *in silico* clinical trials. The fracture healing algorithm presented in this study, demonstrating callus domain independence, is better suited for these applications.

Although the fracture-healing algorithm in this study aligns qualitatively with established literature and quantitatively with corresponding experimental data, the fracture scenario modelled is quite basic: it simulates an ovine metatarsal, which predominantly experiences axial loading (Duda et al., 1997), and the high reported bending and torsional stiffness of the external fixator (Claes et al., 1995) allows for an axisymmetric approximation to reduce computational expense. Realistic clinical fracture scenarios and fixators will require the ability to model non-axisymmetric loading. Therefore, this fracture-healing algorithm should be expanded into a 3D representation to accommodate these expected loads. Modelling in 3D would also enable patient-specific simulations, where bone fragment geometries are identified using CT imaging, and loads are derived from gait analysis and musculoskeletal modelling (Orth et al., 2023). For computational simplicity this first version of the algorithm utilises a static loading case, accepting that this is a slight deviation from the dynamic loading expected in reality.

The tissues modelled in this study are physiologically anisotropic, but were assumed isotropic for simplification, similarly to previous fracture-healing algorithms. The incorporation of anisotropic material models may allow the algorithm to capture more accurately the physiological deformations throughout the healing process. As the present version of the algorithm was able to capture the overall healing progressions and outcomes of the cases simulated nevertheless, modelling anisotropy was deemed unnecessary at this stage. Additionally, the values of material properties used in this study are an uncertainty, reflecting the wide range of values reported from experimental studies and used in numerical simulations. A parametric analysis of the algorithm to material models and material properties used should be considered in future, to understand the sensitivity of the algorithm’s predicted healing outcome to input material properties. A future version of the healing algorithm should consider the results for the sensitivity study and amend the material models of the tissues involved appropriately to reflect reality more accurately.

The significance of principal strains in the fracture-healing process has not been investigated experimentally. Such investigation may help validate the use of minimum and maximum principal strains as mechanical stimuli for the biological fracture-repair process in the fracture-healing algorithm presented here. A previous *in vivo* study on rats has explored various strain measures, including maximum principal strain, in different tissue types during secondary fracture healing, but unfortunately did not report minimum and maximum principal strains to investigate their individual effects on separate ossification processes (Morgan et al., 2010).

## 5 Conclusion

This study presents a novel fracture-healing algorithm which uses minimum and maximum principal strains as the mechanical inputs. The use of these strains allowed for the callus domain independence of the algorithm, without the need to prescribe the temporal-spatial ossification pathway of the callus. Validation was performed against published data from experimental animal studies for cases resulting in both union and, importantly, non-union. The capabilities demonstrated by this algorithm allow for its practical application to pre-surgical planning, novel fracture-fixation device development, rehabilitation-regime management, and non-union risk assessment. Future steps in the development of this algorithm will focus on its expansion to 3D simulation to capture a wider variety of fracture geometries and fixator types. This will allow the comparison between treatments for fracture types which have been insufficiently addressed by traditional clinical trials.

## Data Availability

The original contributions presented in the study are included in the article/Supplementary Material, further inquiries can be directed to the corresponding author.

## References

[B1] Ament C. Hofer E. P. (2000). A fuzzy logic model of fracture healing. J. Biomech. 33, 961–968. 10.1016/s0021-9290(00)00049-x 10828326

[B2] Claes L. Augat P. Suger G. Wilke H. (1997). Influence of size and stability of the osteotomy gap on the success of fracture healing. J. Orthop. Res. 15, 577–584. 10.1002/jor.1100150414 9379268

[B3] Claes L. Recknagel S. Ignatius A. (2012). Fracture healing under healthy and inflammatory conditions. Nat. Rev. Rheumatol. 8, 133–143. 10.1038/nrrheum.2012.1 22293759

[B4] Claes L. Wilke H.-J. Augat P. Rübenacker S. Margevicius K. (1995). Effect of dynamization on gap healing of diaphyseal fractures under external fixation. Clin. Biomech. 10, 227–234. 10.1016/0268-0033(95)99799-8 11415558

[B5] Claes L. E. Heigele C. A. (1999). Magnitudes of local stress and strain along bony surfaces predict the course and type of fracture healing. J. Biomech. 31, 51. 10.1016/s0021-9290(98)80105-x 10093025

[B6] Duda G. N. Eckert-Hübner K. Sokiranski R. Kreutner A. Miller R. Claes L. (1997). Analysis of inter-fragmentary movement as a function of musculoskeletal loading conditions in sheep. J. Biomech. 31, 201–210. 10.1016/S0021-9290(97)00127-9 9645534

[B7] Ghiasi M. S. Chen J. E. Rodriguez E. K. Vaziri A. Nazarian A. (2019). Computational modeling of human bone fracture healing affected by different conditions of initial healing stage. BMC Musculoskelet. Disord. 20, 562. 10.1186/s12891-019-2854-z 31767007 PMC6878676

[B8] Griffin X. L. Costa M. L. Phelps E. Parsons N. Dritsaki M. Png M. E. (2019). Retrograde intramedullary nail fixation compared with fixed-angle plate fixation for fracture of the distal femur: the TrAFFix feasibility RCT. Health Technol. Assess. 23, 1–132. 10.3310/hta23510 PMC6778843 31549959

[B9] Hoffmann W. Feliciano S. Martin I. de Wild M. Wendt D. (2015). Novel perfused compression bioreactor system as an *in vitro* model to investigate fracture healing. Front. Bioeng. Biotechnol. 3, 10. 10.3389/fbioe.2015.00010 25699254 PMC4313709

[B10] Isaksson H. Van Donkelaar C. C. Huiskes R. Ito K. (2008). A mechano-regulatory bone-healing model incorporating cell-phenotype specific activity. J. Theor. Biol. 252, 230–246. 10.1016/j.jtbi.2008.01.030 18353374

[B11] Isaksson H. Van Donkelaar C. C. Ito K. (2009). Sensitivity of tissue differentiation and bone healing predictions to tissue properties. J. Biomech. 42, 555–564. 10.1016/j.jbiomech.2009.01.001 19233361

[B12] Isaksson H. Wilson W. Van Donkelaar C. C. Huiskes R. Ito K. (2006). Comparison of biophysical stimuli for mechano-regulation of tissue differentiation during fracture healing. J. Biomech. 39, 1507–1516. 10.1016/j.jbiomech.2005.01.037 15972212

[B13] Iwaki A. Jingushi S. Oda Y. Izumi T. Shida J.-I. Tsuneyoshi M. (1997). Localization and quantification of proliferating cells during rat fracture repair: detection of proliferating cell nuclear antigen by immunohistochemistry. J. Bone Min. Res. 12, 96–102. 10.1359/jbmr.1997.12.1.96 9240731

[B14] Kohli N. Theodoridis K. Hall T. A. G. Sanz-Pena I. Gaboriau D. C. A. Van Arkel R. J. (2023). Bioreactor analyses of tissue ingrowth, ongrowth and remodelling around implants: an alternative to live animal testing. Front. Bioeng. Biotechnol. 11, 1054391. 10.3389/fbioe.2023.1054391 36890911 PMC9986429

[B15] Lacroix D. Prendergast P. J. (2002). A mechano-regulation model for tissue differentiation during fracture healing: analysis of gap size and loading. J. Biomech. 35, 1163–1171. 10.1016/S0021-9290(02)00086-6 12163306

[B16] Litrenta J. Iii P. T. Mehta S. Jones C. O'Toole R. V. Bhandari M. (2015). Determination of radiographic healing: an assessment of consistency using RUST and modified RUST in metadiaphyseal fractures. J. Orthop. Trauma 29, 516–520. 10.1097/bot.0000000000000390 26165265

[B17] Megafu M. Mian H. Megafu E. Singhal S. Lee A. Cassie R. (2022). The fragility of statistical significance in distal femur fractures: systematic review of randomized controlled trials. Eur. J. Orthop. Surg. Traumatol. 33, 2411–2418. 10.1007/s00590-022-03452-3 36461949

[B18] Morgan E. F. Salisbury Palomares K. T. Gleason R. E. Bellin D. L. Chien K. B. Unnikrishnan G. U. (2010). Correlations between local strains and tissue phenotypes in an experimental model of skeletal healing. J. Biomech. 43, 2418–2424. 10.1016/j.jbiomech.2010.04.019 20546756 PMC2935472

[B19] Nayak G. S. Roland M. Wiese B. Hort N. Diebels S. (2024). Influence of implant base material on secondary bone healing: an *in silico* study. Comput. Methods Biomech. Biomed. Engin., 1–9. 10.1080/10255842.2024.2338121 38613482

[B20] Orth M. Ganse B. Andres A. Wickert K. Warmerdam E. Müller M. (2023). Simulation-based prediction of bone healing and treatment recommendations for lower leg fractures: effects of motion, weight-bearing and fibular mechanics. Front. Bioeng. Biotechnol. 11, 1067845. 10.3389/fbioe.2023.1067845 36890916 PMC9986461

[B21] Pappalardo F. Russo G. Tshinanu F. M. Viceconti M. (2019). *In silico* clinical trials: concepts and early adoptions. Brief. Bioinform. 20, 1699–1708. 10.1093/bib/bby043 29868882

[B22] Pappalardo F. Wilkinson J. Busquet F. Bril A. Palmer M. Walker B. (2022). Toward A regulatory pathway for the use of *in silico* trials in the ce marking of medical devices. IEEE J. Biomed. Health Inf. 26, 5282–5286. 10.1109/JBHI.2022.3198145 35951559

[B23] Quinn C. Kopp A. Vaughan T. J. (2022). A coupled computational framework for bone fracture healing and long‐term remodelling: investigating the role of internal fixation on bone fractures. Int. J. Numer. Methods Biomed. Eng. 38, e3609. 10.1002/cnm.3609 PMC9540005 35485134

[B24] Ren T. Dailey H. L. (2020). Mechanoregulation modeling of bone healing in realistic fracture geometries. Biomech. Model. Mechanobiol. 19, 2307–2322. 10.1007/s10237-020-01340-5 32524288

[B25] Ren T. Inglis B. Darwiche S. Dailey H. L. (2024). Torsion constants and virtual mechanical tests are valid image‐based surrogate measures of ovine fracture healing. J. Orthop. Res. 42, 1810–1819. 10.1002/jor.25836 38491964

[B26] Sarrami-Foroushani A. Lassila T. MacRaild M. Asquith J. Roes K. C. B. Byrne J. V. (2021). In-silico trial of intracranial flow diverters replicates and expands insights from conventional clinical trials. Nat. Commun. 12, 3861. 10.1038/s41467-021-23998-w 34162852 PMC8222326

[B27] Schwarzenberg P. Klein K. Ferguson S. J. Von Rechenberg B. Darwiche S. Dailey H. L. (2021a). Virtual mechanical tests out‐perform morphometric measures for assessment of mechanical stability of fracture healing *in vivo* . J. Orthop. Res. 39, 727–738. 10.1002/jor.24866 32970350

[B28] Schwarzenberg P. Ren T. Klein K. Von Rechenberg B. Darwiche S. Dailey H. L. (2021b). Domain-independent simulation of physiologically relevant callus shape in mechanoregulated models of fracture healing. J. Biomech. 118, 110300. 10.1016/j.jbiomech.2021.110300 33601180

[B29] Shefelbine S. J. Augat P. Claes L. Simon U. (2005). Trabecular bone fracture healing simulation with finite element analysis and fuzzy logic. J. Biomech. 38, 2440–2450. 10.1016/j.jbiomech.2004.10.019 16214492

[B30] Simon U. Augat P. Utz M. Claes L. (2011). A numerical model of the fracture healing process that describes tissue development and revascularisation. Comput. Methods Biomech. Biomed. Engin. 14, 79–93. 10.1080/10255842.2010.499865 21086207

[B31] Steiner M. Claes L. Ignatius A. Simon U. Wehner T. (2014). Numerical simulation of callus healing for optimization of fracture fixation stiffness. PLoS ONE 9, e101370. 10.1371/journal.pone.0101370 24991809 PMC4081589

[B32] Vetter A. Epari D. R. Seidel R. Schell H. Fratzl P. Duda G. N. (2010). Temporal tissue patterns in bone healing of sheep. J. Orthop. Res. 28, 1440–1447. 10.1002/jor.21175 20872579

[B33] Viceconti M. Emili L. Afshari P. Courcelles E. Curreli C. Famaey N. (2021). Possible contexts of use for *in silico* trials methodologies: a consensus-based review. IEEE J. Biomed. Health Inf. 25, 3977–3982. 10.1109/JBHI.2021.3090469 34161248

[B34] Wadhwa H. Salazar B. P. Goodnough L. H. Van Rysselberghe N. L. DeBaun M. R. Wong H.-N. (2022). Distal femur replacement versus open reduction and internal fixation for treatment of periprosthetic distal femur fractures: a systematic review and meta-analysis. J. Orthop. Trauma 36, 1–6. 10.1097/BOT.0000000000002141 34001801

[B35] Warner J. Sexauer J. Scikit-Fuzzy Twmeggs A. Aishwarya U. Castelão G. (2019). JDWarner/scikit-fuzzy: Scikit-Fuzzy version 0.4.2. 10.5281/ZENODO.3541386

[B36] Whelan D. B. Bhandari M. Stephen D. Kreder H. McKee M. D. Zdero R. (2010). Development of the radiographic union score for tibial fractures for the assessment of tibial fracture healing after intramedullary fixation. J. Trauma Inj. Infect. Crit. Care 68, 629–632. 10.1097/TA.0b013e3181a7c16d 19996801

