# Supplementary material for: A novel strain-based bone-fracture healing algorithm is able to predict a range of healing outcomes
Source: Front Bioeng Biotechnol. 2024 Oct 18;12:1477405. doi: 10.3389/fbioe.2024.1477405 (PMC11527658; doi:10.3389/fbioe.2024.1477405)
Supplement: Supplementary file 1 [file DataSheet1.PDF]

Supplementary Material

1 Mesh Convergence Study

The fracture healing algorithm was run with the group B geometry and fixator conditions (1 mm osteotomy, 31% initial IFS). The algorithm was run with callus element edge lengths of 0.5 mm, 0.35 mm, 0.25 mm, and 0.2 mm. The parameter coefficient used for each of these meshes was 0.4, 0.57, 0.8, and 1, respectively. These coefficients were calculated by inversely scaling the coefficient to the element edge length; for example, from the 0.5 mm edge length to the 0.2 mm edge length, the coefficient is scaled by 0.5/0.2 = 2.5, yielding 0.4 * 2.5 = 1. The temporal smoothing parameter N for each of these meshes was 10, 7, 5, and 4, respectively. These temporal smoothing parameters were calculated by directly scaling the parameter to the element edge length, and rounding to the nearest whole number.

The IFMs during the healing process are shown in Figure S1 for each of the callus mesh element edge lengths. The mesh was converged with an element edge length of 0.35 mm.

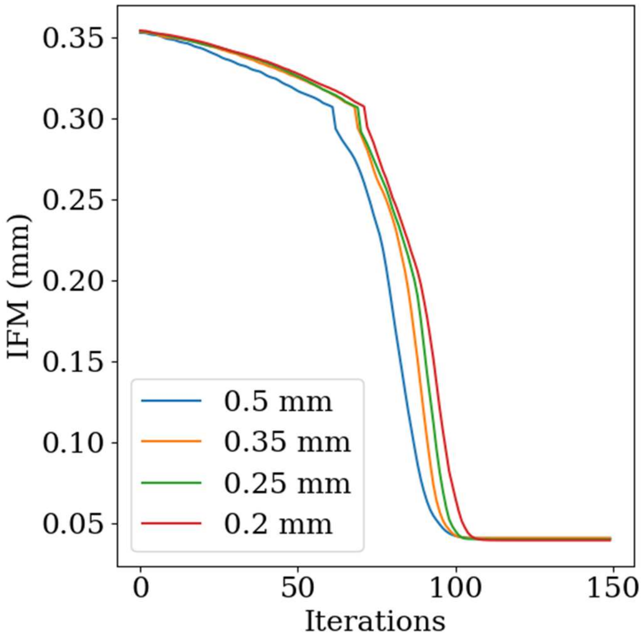

Supplementary Figure 1. Temporal evolution of IFM across the healing process for group B using a callus mesh element edge length of 0.5 mm, 0.35 mm, 0.25 mm, and 0.2 mm.
